# La violencia obstétrica como injusticia epistémica: el parto en disputa

**DOI:** 10.18294/sc.2023.4464

**Published:** 2023-10-03

**Authors:** Ester Massó Guijarro

**Affiliations:** 1 Doctora en Filosofía y Antropología. Profesora titular de Filosofía Moral, Departamento de Filosofía I, Universidad de Granada, Granada, España. ester@ugr.es Universidad de Granada Departamento de Filosofía I Universidad de Granada Granada Spain ester@ugr.es

**Keywords:** Violencia Obstétrica, Bioética, Antropología Cultural, Feminismo, Epistemología, Salud Pública, Violence, Bioethics, Cultural Anthropology, Feminism, Epistemology, Public Health

## Abstract

Este artículo aborda en términos teóricos la cuestión de la violencia obstétrica como injusticia epistémica, con especial énfasis en las perspectivas que propone la filosofía fenomenológica feminista, desde el encuadre general de la bioética narrativa y la lucha por los derechos sexo-reproductivos. En la primera parte, se aborda el concepto de violencia obstétrica, enfatizando el carácter pionero de América Latina en su acuñe y reconocimiento, así como en su aplicación empírico-hermenéutica. En la segunda parte, se examina cómo el concepto de violencia obstétrica ha sido analizado a través del prisma de la injusticia epistémica (en sus dos versiones: testimonial y hermenéutica), lo que ha supuesto un avance significativo en su comprensión sistémica y en su carácter biopolítico. El artículo concluye sobre la plena pertinencia empírico-teórica del término, en tanto concepto filosófico denso, pese a la controversia existente entre la clase biosanitaria (especialmente médica) y la reclamación ciudadana.

## Abstract (en)

This article theoretically frames the issue of obstetric violence as epistemic injustice, drawing heavily from feminist phenomenological philosophy, within the general framework of narrative bioethics and the fight for sexual-reproductive rights. The first section deals with the concept of obstetric violence, emphasizing Latin America’s pioneering role in its coinage and recognition, as well as its empirical-hermeneutical applications. In the second section, consideration is given to how the concept of obstetric violence has been analyzed through the lens of epistemic injustice (in its two versions: testimonial and hermeneutic), which has signified major progress in its systemic understanding and its biopolitical nature. The article’s conclusions highlight the full empirical-theoretical relevance of the term, as a thick philosophical concept, despite existing tensions between the biosanitary (especially medical) sector and citizen demands.

## INTRODUCCIÓN

### La “patata caliente” de la violencia obstétrica

Muchas mujeres en todo el mundo experimentan un trato irrespetuoso, abusivo o negligente durante el parto.^1^

### “A las barricadas”: el interregno de la violencia obstétrica

*Se me hacía callar si gritaba fuerte. Te resignas a pensar que las cosas son así*. M.^2^

La crisis consiste precisamente en el hecho de que lo viejo muere y lo nuevo no puede nacer: en ese interregno se verifican los fenómenos morbosos más variados.^3^

En 2007, Venezuela lanzó un órdago al mundo aprobando la primera ley contra la violencia obstétrica. Toda ficción es una fundación y también lo son las cartografías jurídicas: ficciones cognitivas, como cualquier constructo en aras de la emancipación humana. 

Este artículo trata, entre otras cosas, de avizorar una mirada reflexiva sobre cómo esta inmensa y necesaria ficción ha devenido, cada vez más, escoplo epistémico y bandera crítica, una auténtica barricada instituida en la realidad empírica de tantas personas subalternas (parturientas y paridas) en todo el mundo, y de cómo se ha *fundado* una nueva realidad. Aún estamos en ese interregno que tan bien describió Antonio Gramsci para las crisis^3^, aún tenemos que denunciar *negacionismo* de la violencia obstétrica, y aún es necesario un trabajo como este y el de tantas otras.

Venezuela lanzó ese órdago al mundo en 2007^4^. Lo lanzó al mundo, el mundo de las mujeres, de las madres, el mundo de la vida de Popper, el mundo de la obstetricia, también, entre otros. Y al mundo de la filosofía. 

Tras Venezuela, un rosario de países latinoamericanos, en un proceso absolutamente pionero que amerita un reconocimiento genuino, fueron generando y aplicando leyes en contra de la violencia obstétrica. Había nacido un nuevo mundo, todavía embrión, sin embargo, en el viejo que no acababa de perecer. Por eso vamos a tratar aquí ese *interregno* de la violencia obstétrica, en tanto concepto hermenéutico y denuncia política.

El Comité de Naciones Unidas para la Eliminación de la Discriminación contra la Mujer (CEDAW), considerado el tratado de derechos humanos más importante para las mujeres, condenó a España en 2020 “por no actuar de manera diligente para proteger los derechos de una mujer y su hija a una atención obstétrica de calidad y libre de violencia”^5^. 

Ante el recrudecimiento de las querellas ciudadanas y movimientos denunciantes de violencia obstétrica, el presidente del Colegio de Médicos de Madrid, Martínez Sellés, afirmó en un programa de televisión nacional en horario de máxima audiencia: “Me preocupa que se pueda generar una situación de desconfianza médico-paciente […] Creo que es un error que esto nos lleve a hablar de violencia obstétrica” ^(^^6^. En otras palabras, este representante público y tomador de decisiones negó sin ambages el concepto de violencia obstétrica. Aquello, por supuesto, no quedó ahí, sino que terminó por cristalizar en un comunicado oficial crítico de la Comisión de Deontología de aquel Colegio de Médicos de la capital de mi propio país^7^^,^^8^.

De hecho, en España en particular y en Europa en general, es muy candente el debate *incluso* sobre la mera existencia (el debate *ontológico*, podríamos decir): si *existe* la violencia obstétrica, con ese nombre, defendida ardientemente por asociaciones subversivas y radicalmente feministas como “El parto es nuestro”, que la sancionó en 2016 como violencia estructural y de la que nació también el actual Observatorio de la Violencia Obstétrica en España; una forma de violencia específica contra mujeres y criaturas reconocida ya por la CEDAW de la ONU^9^. 

Sin embargo, y como ha sido ya señalado también desde la ciencia:

España parece tener un grave problema de salud pública y de respeto a los derechos humanos en la violencia obstétrica. Ofrecer información a las mujeres y solicitar su consentimiento informado es una práctica escasa en el sistema sanitario, por lo que es necesario reflexionar profundamente sobre las prácticas obstétricas y solicitar el consentimiento informado a las mujeres en España” [Traducción libre de: *Spain seems to have a serious problem with public health and respecting human rights in obstetric violence. Offering information to women and requesting their informed consent are barely practiced in the healthcare system, so it is necessary to profoundly reflect on obstetric practices with, and request informed consent from, women in Spain*].^10^

Todo ello, pese a que

…en España las prácticas que definimos como constitutivas de violencia obstétrica se encuentran prohibidas, en cuanto suponen la vulneración de derechos fundamentales reconocidos en la Constitución Española. Estas tienen a que ver con la integridad física y moral (artículo 15), la libertad personal (artículo 17) y la intimidad (artículo 18).^11^

Por otro lado, desde los análisis feministas se ha investigado cómo la violencia obstétrica se ha exacerbado en la pandemia^12^^,^^13^^,^^14^^,^^15^^,^^16^^,^^17^^,^^18^^,^^19^, que se ha tornado una excusa para la cancelación de derechos fundamentales. 

A la misma definición de violencia obstétrica, como señala Castrillo^20^ con gran lucidez, subyace “un campo de disputa por las legitimidades (de su definición), en una interrelación constante entre las nominaciones objetivas y las significaciones subjetivas sobre ciertas prácticas obstétricas, definidas como violentas”. Resulta, así, fundamental problematizar la definición misma de la violencia obstétrica, que nace en ciertas prácticas y relaciones en la atención sanitaria de partos, ese “trato deshumanizado, irrespetuoso, jerarquizado, y la atención insuficiente en el ámbito del parto”^21^.

Y es que la violencia obstétrica tiene que ver con la política, pero también con el *nombrar*, con el título que le damos a las cosas (las realidades, los fenómenos) y, por ende, cómo las *reconocemos*. Tiene que ver, entre otras cosas, con el capital epistémico-político de las personas sujetas de derechos, a lo que apunta la médula espinal de esta investigación. Por fortuna son ya legión los estudios, desde ámbitos y perspectivas muy dispares, sobre violencia obstétrica, de modo que mi trabajo aquí pretende algo muy concreto frente a todo ese maremágnum: mi perspectiva sobre la cuestión busca aportar algunos elementos fundamentales que se están trabajando en filosofía y ciencias humanas y sociales. 

Es evidente el cariz de *concepto en disputa* que la violencia obstétrica ha alcanzado en nuestros días. Muy recientemente, se está comenzando a vincular con la noción de injusticia epistémica, casi como un ejemplo paradigmático de ello: en ello se centra este artículo, dado que el análisis de la violencia como injusticia epistémica, aunque muy joven, ya presenta relevantes exponentes. La relación de ambos conceptos es poderosamente intuitiva, a mi entender: resultaba inevitable la unión de ambos términos teóricos, controvertidos de por sí: uno pura guerrilla, el otro puro éxito y aplicación a campos diversos^22^.

Mi trabajo, pues, profundiza en la ilación de ambas nociones, en esa implementación de la lente de la injusticia epistémica a la violencia obstétrica, con todo su contencioso conceptual y práctico, pergeñando esa mirada poliédrica sobre el concepto en disputa y, sobre todo, en el interregno en el que se halla. Para ello, entiendo que su vinculación hermenéutica crítica con la noción de injusticia epistémica, a tenor además de su aproximación filosófica-fenomenología feminista, resulta crucial y, a mi criterio, de los más iluminadores hasta la fecha. De todo ello tratará de dar cuenta este artículo: tras esta introducción (que contiene, a continuación, una sucinta nota sobre episteme y métodos), subsigue un segundo epígrafe acerca del concepto de violencia obstétrica, a modo de marco teórico (no exhaustivo, ya que ello no es aquí el objetivo principal) para pasar finalmente a un tercer y principal punto del trabajo, que profundiza en la relación de la violencia obstétrica con la injusticia epistémica de forma más desagregada (a su vez en dos subsecciones). 

## NOTA SOBRE EPISTEME Y MÉTODOS

### Bioética narrativa, etnografía, autoetnografía

*No me dejaban comer nada ni beber agua. Recuerdo eso de una forma casi física. La sed. Esa sed intensa que sientes que se te va a pegar la lengua al paladar y que tienes la boca como corcho. Eso y el dolor. El dolor cada vez se hizo más insoportable* […] *Fue horrible para mí, el sentirme ignorada, que hacían conmigo lo que querían* […] *Me sentía como una atracción de feria*. A.I.^2^

En este artículo es capital el enfoque de la bioética narrativa^23^, complementariamente con la etnografía y la autoetnografía como miradas-métodos, y aunque no se trata de un trabajo propiamente empírico, considero que contribuye al marco genérico de la bioética^24^ y la salud colectiva, y a la perspectiva multi-inter-transdisciplinar y de los derechos humanos.

En cuanto a la bioética narrativa, la entendemos en el paradigma narrativo general como parte del “grupo de aproximaciones éticas que exploran el potencial de la experiencia narrada, la capacidad imaginativa y los contextos de interpretación”^25^; ello, complementariamente a la filosofía de la narración^26^, desarrollada por la feminista contemporánea Adriana Cavarero. Las citas que abren la mayoría de las secciones de este texto son ejemplo de ello: se trata de testimonios directos, públicamente compartidos, de madres que han padecido violencia obstétrica, en reconocimiento de su autoridad moral.

Estas miradas son cruciales y estructuradoras en mi investigación genérica ya de tres lustros sobre maternidades, elaborada desde una perspectiva tanto filosófica como antropológica y etnográfica en lo metodológico; todo ello, es la base *experiencial* de este artículo, aunque este constituya un trabajo independiente y autónomo, eminentemente analítico y fenomenológico. Dentro de esta óptica etnográfica -como se verá, la etnografía es a menudo el método preferido en muchas de las investigaciones aquí citadas^27^, aunque no provengan directamente de la antropología-, la *autoetnografía* ha sido y es asimismo un método investigador crucial para mi trabajo (desde 2008, y en tanto que madre lactivista de dos criaturas e investigadora, realizo una incesante actividad de participación y observación en distintos grupos, formales e informales, y espacios sociales y científicos de todo tipo, sobre experiencias vinculadas con el matriactivismo). Ello viene avalado por las publicaciones de otras colegas en la misma línea en revistas referenciales, que apelan y aluden a sus propias experiencias autobiográficas y con talante autoetnográfico, como el caso paradigmático (entre otros) de Sara Cohen Shabot. Esto es parte también de lo que a menudo se llama pesquisa militante, entre otros tantos modos posibles de denominar la imbricación de lo personal (y carnal, y sexual) y lo político y, sobre todo, su legitimación también (o muy especialmente) en la academia.

## LA VIOLENCIA OBSTÉTRICA: CARTOGRAFÍA DE UN CONCEPTO EN LLAMAS

“Las verdades desnudas ofenden a las mentiras vestidas” (Eduardo Galeano).

Como señalamos al inicio, una de las grandes controversias sobre la violencia obstétrica tiene que ver con las cuestiones *ontológico-nominativas*: ¿cómo nombrarla?^28^. Decir “violencia” ligada a la obstetricia *incomoda*, especialmente a la clase médico-sanitaria. Es cierto que la CEDAW no usó el término literalmente “violencia obstétrica” en la sentencia antes aludida, sino “violencia ligada a las prácticas obstétricas”, lo que en realidad es lo mismo en un sentido lógico y expresivo. 

Sin embargo, el lenguaje debe servir para resolver problemas, no para crear más o empeorar los ya existentes. ¿Hay que buscar otro nombre, o acaso suavizar este?^29^. ¿Menos lesivo? ¿Lesivo para quién? Porque las diadas madre-bebé ya llegan lesionadas a esta palestra dialéctica, mientras nosotras debatimos. 

Por otro lado, el uso inflacionario y excesivo de un concepto lo puede hacer difuso, inútil incluso. Cuando algo empieza a servir para todo (¿ser un comodín?) deja de servir para nada. ¿Está sucediendo eso, tal vez, con la violencia obstétrica? ¿Cualquier cosa -cualquier mala práctica o error- es violencia obstétrica? ¿Estamos llamando violencia a lo que puede ser simplemente una mala praxis, como la hay en tantos otros espacios? ¿O incluso lo estamos usando mal, y decir “violencia” puede suponer una intencionalidad que en realidad no es atribuible a la profesión sanitaria, en cuyo origen y sentido está la sanación? Esto último es de hecho lo que se suele aducir por parte de la clase biosanitaria para criticar el concepto^30^; argumento que, sin embargo, me resulta epistémicamente insatisfactorio: es como decir que no podemos usar el término violencia machista o de género en el ámbito de una relación de pareja, ya que el origen y sentido de una pareja es cuidarse y quererse.

Todas estas son, en todo caso, seguramente, preguntas legítimas. Veamos un poco de historia sobre la cuestión, sobre cómo se define la violencia obstétrica (primero, el *nombrar*) y sobre cómo se combate (segundo, el *subvertir*), a través de la legislación y a través de su concienciación en la esfera pública, es decir, con un alcance multinivel^31^.

La evolución *de* y *hacia* el concepto de violencia obstétrica sin duda ha sido y es un ejemplo magnífico, paradigmático, de la teoría y la práctica de las palabras, de la insurrección pragmática (retomaremos mi idea de la insurrección conceptual al final), de su avance en los programas políticos y en la calle, en las auténticas barricadas que suponen esos movimientos de madres y criaturas, genuinamente contestatarios y transformadores (mucho más que otros tantos movimientos sociales más estudiados y visibilizados como tal), que han reclamado contra la violencia obstétrica, que les atañe de modo fundamental; movimientos con un carácter de marea social, profundamente políticos, desde una perspectiva libertaria de la acción política^32^.

Un dato que siempre me ha resultado fundamental sobre la violencia obstétrica (por ello quise enfatizarlo al inicio de este texto) es dónde se origina el concepto: en América Latina, siendo Venezuela el primer país que legisla como tal^4^^,^^33^^,^^34^^,^^35^. De hecho, países como Argentina destacan claramente en su aporte pionero al debate internacional, especialmente a través de su implementación del Observatorio de Violencia Obstétrica^34^, mientras que, en Europa, hasta muy recientemente ningún país ha aprobado legislación específica, pese al alto nivel de debate y enconamiento social entre las discusiones académicas, de organizaciones civiles y diversos movimientos ciudadanos^36^. Y ello no obedece, por cierto, como a menudo se ha argüido^34^^,^^35^, a que en los países “ricos” no haya violencia obstétrica.

Solo muy recientemente en Europa, de manera precursora y con muchísimas reticencias por parte de la clase médica, la región de Cataluña en España ha legislado como tal, denunciando que la violencia obstétrica puede afectar la salud física y mental, así como la salud sexual y reproductiva, matizándola además como el “prevenir o dificultar el acceso a información veraz, necesaria para la toma de decisiones autónoma e informada”^37^, destacando igualmente la formalización del Observatorio de la Violencia Obstétrica, arriba citado.

Dicha definición es esencial para los intereses reflexivos de mi trabajo, porque alude específicamente a la *información*: al derecho a la información veraz, necesaria, y a su hurto o no facilitación como germen de aquella violencia. Esto se vincula específicamente con el concepto de injusticia epistémica, una forma singular y genuina de injusticia que tiene que ver con la información (su posesión, su procesamiento) y la cognición, justamente, y cómo las reconocemos o no.

Como señala la investigación referencial de Patrizia Quattrocchi^34^, la violencia obstétrica puede darse tanto por *defecto* como por *exceso* de atención sanitaria, y ambos casos ocurren a menudo en todos los continentes. Es cierto que en países de muy bajos ingresos suele ser más frecuente la violencia obstétrica por *falta* de medios suficientes, pero ambas versiones de esta son dos rostros de lo mismo (y probablemente, todo ello, un rasgo elemental de la deriva antropocénica a un mundo hiper-tecnologizado): la inadecuación en la atención de un proceso universal crucial, con alcance humano generalizado. *Nacer* -como morir^27^- afecta a toda la comunidad humana, no solo a las madres parturientas, que ya de por sí son suficiente motivo político y ético, por supuesto.

Como apunta Quattrocchi^34^, la violencia obstétrica constituye un nuevo término legal destinado a *proteger* a las mujeres durante el parto; considero que es imperativo incluir también en esa perentoriedad de la protección también a las *criaturas*, los bebés, en tanto muchas de las prácticas negativas para las parturientas lo son a la vez para el neonato, y sus intereses confluyen en todos los sentidos, hay una comunidad de intereses y en un proceso de parto no hay aún dos entes o individuos diferenciados del todo (no en un sentido cartesiano puro, ni siquiera en un sentido biológico y fisiológico). En la legislación específica contra la violencia obstétrica, además, destaca el elemento de que se la considera un tipo de violencia *basada en el género* (reconocido por la ONU, como arriba señalábamos) y una violación de los derechos humanos relacionados con la salud reproductiva.

La necesidad de un marco teórico que ilustre la complejidad del fenómeno ha sido ya largamente reconocida y cuenta con interesantes desarrollos. Bellón^33^ destaca las aportaciones de la crítica biopolítica y feminista (que retomaré más adelante), enfatizando el elemento que el control de la asistencia médica en el parto y, por ende, la lucha por la posesión del conocimiento legitimado, generan en la obstetricia contemporánea^33^.

En el estudio de Smith-Oka *et al*.^38^, que realizaron una investigación etnográfica en México y Sudáfrica, encontramos dos argumentos centrales sobre la violencia obstétrica: en primer lugar, las desigualdades estructurales en diversos sitios globales están principalmente vinculadas al género y conducen a patrones similares de violencia obstétrica; en segundo lugar, la etnografía es un poderoso método para dar voz a las historias de las mujeres. Esto nos lleva nuevamente a la cuestión de la narración y la filosofía de la narración^26^, precisamente para subvertir esa injusticia epistémica, testimonial y hermenéutica, subversión que tiene mucho de restañar heridas, de dar voz, de escuchar, de respetar esa voz secularmente desconsiderada^28^. Conectar estos dos argumentos (la similitud contextual y la etnografía) es un modelo temporal, según sus autoras, para comprender cómo las mujeres de todo el mundo esperan, experimentan y responden a la violencia obstétrica, es decir, antes, durante y después del encuentro. Todo ello, a su vez, requiere una articulación jurídica, la necesidad de un cambio de paradigma en el ámbito jurídico, que constituye una cartografía reglamentaria de los derechos^11^.

Por su parte, los abordajes de corte más descriptivo, siguiendo el estilo etnográfico de Smith-Oka^38^, son precisos para mostrar, para nombrar, para encarnar (*embodiment*, en su término clásico en inglés). Medeiros *et al*.^39^ analizan en su estudio cualitativo la violencia obstétrica y sus diversas expresiones desde la perspectiva de la mujer puérpera en el contexto brasileño, y concluyen su recurrencia en la atención hospitalaria, que se refleja además en desigualdades y opresión en las relaciones de género y entre profesionales y usuarias de los servicios de salud. Apoyan, en fin, la necesidad de su mayor visibilidad, a través también de una formación estructural multinivel sobre el tema^31^.

Margarita^40^ incide también en el análisis de la violencia obstétrica como violencia de género, desde un estudio etnográfico de la violencia asistencial en el embarazo y el parto en España, y de la percepción de usuarias y profesionales. Arguedas Ramírez^41^, por su parte, analiza la violencia obstétrica y el saber/poder obstétrico en Costa Rica, desde la perspectiva crítica de la epistemología feminista y el feminismo decolonial. Llobera *et al*.^21^ aplican el enfoque fenomenológico en su estudio cualitativo para el ámbito español, en el que se destacan igualmente una serie de investigaciones referenciales^28^^,^^42^^,^^43^^,^^44^.

En su análisis, Castrillo^20^ propone dos ejes de trabajo interrelacionados: por un lado, la aplicación de un modelo de análisis socioantropológico de la violencia para el estudio de la violencia obstétrica; por otro lado, el cruce entre definiciones objetivas (académicas, legales, políticas) y subjetivas, en aras de abordar dicha problematización. Y es obvio que “el cambio en las sensibilidades sociales sobre diversas formas de violencia (que generó cambios en las legitimidades) explica la actual discusión sobre la definición de la violencia obstétrica”^20^. En otras palabras, lo que antes no era visibilizado ni asumido (*comprendido*) como violencia (como ha sucedido en otros tantos terrenos históricos en los que faltaba la fundación de un concepto analítico), ahora sí lo es… y, por tanto, también sucede en la aplicación del concepto a la propia obstetricia. Esto será crucial, como veremos, en su implementación en el campo de la filosofía y hermenéutica, en la ardorosa encrucijada entre violencia obstétrica e injusticia epistémica.

Sadler *et al*.^45^ han avanzado igualmente de forma notoria en la comprensión y descripción de la dimensión estructural de la violencia obstétrica, tanto en términos generales como en su realidad en Chile. Pese al ímprobo tratamiento de la cuestión en las últimas décadas, persisten tasas excesivas de intervenciones médicas y la falta de respeto hacia las mujeres durante el parto, como consecuencia de aquella violencia estructural^45^. Ello supone, entre otras cosas, la necesidad y la importancia del *activismo* por el parto respetado.

Esta cuestión estructural, que sucede porque el sistema está enfermo, tal como señalan tantas autoras^30^, y que es aplicable a otros contextos de biopolítica corporal como la hipogalacia social en tanto que sindemia^14^, resulta central y será abordada más adelante en mi argumentación.

## LA VIOLENCIA OBSTÉTRICA COMO INJUSTICIA EPISTÉMICA: LA INTERSECCIÓN INCENDIARIA

*Quizás la palabra “violencia”* [obstétrica] *sea fea. Y difícil de admitir por los médicos; es normal que no guste. Pero a veces hay que dar un golpe sobre la mesa, despertar conciencias y cuando deje de ser una realidad escondida, entonces dejar de usar ese término*. Irene G.^46^

### Violencia (obstétrica) e injusticia (epistémica): el magma de la biopolítica

*Las consecuencias de un parto no respetado son tremendas. Me habéis destrozado, pero te piden que te consueles con que “tienes un bebé precioso”*. M.^2^

Como se ha tratado de dar cuenta en el epígrafe anterior, los abordajes desde distintas disciplinas y miradas sobre violencia obstétrica son numerosos y plurales. Las reivindicaciones contemporáneas en torno al concepto (en disputa) de violencia obstétrica, así como sus implicaciones, deben armarse desde distintos prismas epistémicos y ámbitos de conocimiento, ya que las propias realidades que se hallan en la base de todo ello son esencialmente híbridas, en tanto *bioculturales*.

 Sin embargo, como estudiosa de la filosofía y la antropología, lo que me parece fundamental aquí y deseo teorizar es el camino de analizar la violencia obstétrica como una *forma paradigmática de injusticia epistémica*, lo cual nos ayudaría también a comprender el porqué de su persistencia, en función de aquel carácter estructural que acabamos de señalar. Se trata, así, de una cuestión sistémica, de magma biopolítico^47^. Barros y Guimarães^48^ ya aplican esta óptica en un perspicaz análisis del caso de Brasil, siempre desde la injusticia epistémica, centrándose en el análisis de relatos de casos de violencia obstétrica, para enfatizar el aspecto epistémico de tales violencias, y cuán relevante sería un *cambio en la distribución de la credibilidad* para el afrontamiento de la violencia obstétrica misma.

“Injusticia epistémica” es una noción publicada por primera vez como tal en 2007 por Miranda Fricker^49^ y, ya desde su albor, ha reproducido notables vástagos hermenéuticos, vástagos mestizos e insólitos: su alcance y su aplicación hoy han rebasado fronteras de todo tipo^13^^,^^50^. Fricker^49^ señala también las desventajas cognitivas, el vacío de recursos hermenéutico, el concepto de marginación hermenéutica o el de desigualdad hermenéutica difícil de detectar, como algunos otros subconceptos cruciales.

Una injusticia epistémica (en sus dos versiones, *testimonial* y *hermenéutica*) se produce cuando se anula la capacidad de una persona para dar sentido a sus experiencias sociales y transmitir conocimiento^13^. Esto ha sucedido a gran escala, a escala sociocultural, por ejemplo, cuando decimos que en un genocidio concurre también un epistemicidio, la aniquilación y el asesinato de formas específicas de conocimiento y ser culturales. 

En esencia, Fricker^49^ analiza y hace visible el error que se comete -y sus consecuencias- cuando se desacredita el discurso de una persona por causas ajenas a su contenido, y más bien relacionadas con su condición personal o grupal, y la desconsideración sistémica a esa condición. Si esta anulación ha sucedido en algún grupo social o contexto de forma paradigmática (del todo acrítica y normalizada hasta fechas recientes, como hemos visto) es, proverbialmente, el ámbito obstétrico de la atención al parto y, concretamente, las voces de las mujeres parturientas, embarazas y puérperas (y subsidiariamente con ellas, por supuesto, la voz, aún sin forma, de las criaturas). La obstetricia y la ginecología, de hecho, han sido dos de los patriarcados más poderosos, porque se han vinculado con el aspecto más sexual (y sexualizado) del cuerpo y las capacidades fisiológicas de las mujeres, a saber, su reproducción, su sexualidad, y la dimensión corpórea en todo ello.

Hay aquí también pues un reclamo de la importancia de la *narración* en la violencia obstétrica; Cavarero^26^ (arriba señalada en conjunción con el enfoque de la bioética narrativa^23^^,^^25^ habla, en su filosofía de la narración, del propio relato como creador de la propia vida. Así, podemos vincular todos estos enfoques en el camino de revocar esta injusticia epistémica tan dolosa, y en su aplicación a una mirada feminista a los derechos sexuales y reproductivos.

La injusticia epistémica es el nombre genérico, a su vez, para dos formas concretas o desagregadas, a saber, la injusticia *testimonial* y la injusticia *hermenéutica*. Vamos a ver qué significan y por qué ambas son plenamente aplicables a la violencia obstétrica, cada una en su especificidad.

La injusticia *testimonial* se produce “cuando los prejuicios llevan a un oyente a otorgar a las palabras de un hablante un grado de credibilidad disminuido”^49^; es decir, nos creemos *menos* lo que expresa una determinada persona y le damos menos importancia porque pensamos que no sabe muy bien lo que dice. Es el caso, por ejemplo, de cuando una madre parturienta profiere una preferencia determinada, antes incluso del parto, y no es respetada, como encontramos sistemáticamente aducido en notorios ejemplos en todos los estudios de corte empírico que hemos citado y revisado arriba.

La injusticia *hermenéutica*, por su parte, se produciría en una fase anterior, “cuando una brecha en los recursos de interpretación colectivos sitúa a alguien en una desventaja injusta en lo relativo a la comprensión de sus experiencias sociales”^49^. Por ejemplo, cuando una parturienta desconoce que la maniobra Kristeller ya no está indicada (lo desconoce porque no tiene por qué ser experta en estas cuestiones), y llega incluso a estar agradecida cuando le rompen una costilla al aplicar este procedimiento, bajo el supuesto de salvar a su bebé. O, por ejemplo, cuando otra madre interrumpe la lactancia porque el pediatra le dice que tiene que tomar un fármaco que es incompatible (no siendo así). En este sentido, cabe destacar, por ejemplo, la magnífica iniciativa e-lactancia.org, que nació para combatir esto, y justo es reconocer que fue un pediatra, Paricio Talayero, quien lo creó; es decir, cuando hablamos de esa “clase médica” no estamos pretendiendo en absoluto una homogeneidad ni tratando de polarizar innecesariamente el debate. Insisto: todos los estudios empíricos citados aluden ejemplos significativos al respecto, no se ha abundado en la descripción de las formas de violencia porque no es el objetivo de este artículo, y se hallan sobradamente descritas en los múltiples trabajos citados.

Hemos pues de entender la violencia obstétrica como paradigmática, en tanto biopolítica, en el sentido del concepto foucaultiano, y de biopoder^51^, propia de un sistema biopolítico androcéntrico o patriarcal. 

Finalmente, la *injusticia* epistémica es una noción que tiene que ver con el reclamo de *justicia*, como es evidente. La justicia es precisa para subvertir ese mundo donde la violencia obstétrica sucede de forma sistémica, incluso siendo *buenas* las personas que la ejercen (se retomará este asunto al final), pero una justicia entendida también como cuidado, o en relación con un paradigma de cuidado, y no tanto en un sentido retributivo. Seyla Benhabib^52^, entre otras, ha argumentado la necesidad de incluir la lógica de la ética del cuidado (siempre tradicionalmente más vinculada a lo femenino) dentro de las éticas de la justicia (siempre, hasta ahora, más tradicionalmente vinculada a lo masculino).

### La violencia obstétrica como injusticia epistémica y la fenomenología feminista: los cuerpos activos y (re) productivos de las mujeres

*Me sentí invisible, ninguneada e infantilizada. Hacían comentarios desagradables o hablaban de sus cosas. Me hicieron montones de tactos, maniobra Kristeller y episiotomía*. R.^2^

Esta sección se centra específicamente en el análisis de la bibliografía concreta hallada, catalogada y revisada que relaciona explícitamente violencia obstétrica con injusticia epistémica desde distintos ámbitos y disciplinas. Especialmente, es de interés para nuestra perspectiva la elaborada en los últimos años desde la filosofía y los estudios de las mujeres por la académica israelí Sara Cohen Shabot, con una perspectiva -entre otras- *autoetnográfica* que yo abrazo especialmente. En su trabajo, Cohen habla abierta y literalmente de sus partos y, por ende, de su propia experiencia como fuente de conocimiento. Yo misma, a menudo, en mis trabajos sobre lactancia -y por ello también de parto, la lactancia se liga necesariamente con el embarazo y con él, con las experiencias fenomenológico-biológicas de ambos-, me he refrenado a la hora de hablar explícitamente de ellos, así que supone una alegría reconfortante, como investigadora, encontrarme los maravillosos escritos de Shabot.

Me interesa especialmente también porque es de las pocas autoras (si no la única) que desde la propia *filosofía fenomenológica* aborda así esta cuestión (los estudios más numerosos hasta ahora provienen de otras miradas) y, sobre todo, porque lo afirma como *feminista*: en un tiempo en que el término “feminismo” está tan deconstruido y reconstruido, poblado de hermenéuticas incluso opuestas entre sí, aquejado de cosméticos ideológicos a veces nada inocuos (y desde luego nada efectivos), y también de corsés normativos que resultan casi escolásticos en otros casos, me parece de singular aliciente que una autora afirme su reflexión fenomenológica como feminista *porque lo hace desde el cuerpo de las mujeres*, *y mujeres parturientas*: el cuerpo que *pare (re) produciendo* otros seres humanos. 

Shabot^53^^,^^54^ analiza, pues, la violencia obstétrica, asumiéndola sin ambages, en primer lugar, a modo de violencia estructural de género que compromete la propia agencia y la autonomía corporal, en la forma de violencia psicológica y física, por parte del personal médico hacia las mujeres parturientas. Como bien señala esta autora, hasta ahora el fenómeno ha sido investigado sobre todo por las ciencias sociales y de la salud (como ha sucedido también en gran medida con los procesos reproductivos en general), pasando desapercibidas ciertas cuestiones teórico-conceptuales cruciales para aprehenderlo. Shabot enfatiza especialmente el elemento de “vulnerabilidad encarnada” en el que se perpetra la violencia obstétrica, que no ha sido reconocido y que, por ello mismo, podría ser destructivo de la subjetividad en tanto que no permite el apoyo y destruye las relaciones (entre las mujeres, sus cuerpos *vividos* y su entorno), así como la interdependencia. 

En su análisis, Shabot echa mano precisamente del enfoque beauvoiriano sobre el sujeto encarnado o incorporado, y esencialmente construido fenomenológicamente a través de sus relaciones; esa comprensión ayudará a entender el genuino daño que sucede en la violencia obstétrica:

El cuerpo oximorónico del parto es notablemente susceptible a la violencia principalmente porque no está solo: porque […] nacemos con otros. [Traducción libre de: *The oxymoronic body of childbirth is notably susceptible to violence mainly because it is not alone: because we* […] *birth with others*].^53^

Shabot^53^^,^^54^ argumenta también la cuestión crucial a través del concepto de injusticia *hermenéutica*: muchas mujeres carecen en efecto de recursos epistémicos que les permitirían *reconocer* como violentas ciertas prácticas durante el parto (por ejemplo, los tactos vaginales, habiendo sido su utilidad ya cuestionada hasta por la OMS y en tanto que supone una técnica claramente innecesaria e invasiva de la intimidad), una falta de recursos que obedece al patriarcado (el gineco-obstétrico y el general), vinculado con la disponibilidad sexual y la mercantilización, que normalizan aquella violencia. Para su mejor comprensión fenomenológica, Shabot hace uso de la distinción de Butler entre “reconocimiento” y “aprehensión”, que ayuda a comprender por qué no son *reconocidos* a menudo como violentos ciertos actos, aunque sí sean *aprehendidos* (y, por ende, sufridos) como tal por los propios sujetos (y las altas tasas de neurosis y depresiones posparto son indicadores de ello, entre otras, sin olvidar la afectación también de las propias profesionales^55^.

Otro asunto fundamental es el del rol de la *vergüenza* (de género, “*gender shame*”) en relación con la violencia obstétrica^56^. En su estela del análisis filosófico de la obstetricia con perspectiva feminista y de género, las autoras destacan cómo la vergüenza, entre otros factores, opera también para contribuir a que la violencia obstétrica suceda de forma normalizada: cómo esta se perpetúa y expande a través de mecanismos de culpabilización para paralizar a las mujeres en sus decisiones en el momento del parto, tornándolas más pasivas y, por ende, menos capaces de afrontar y combatir aquella violencia. Esto sucede de forma específica, por ejemplo, con madres que reclaman un parto más humanizado, y son amenazadas o asustadas de formas diversas por el personal médico bajo la forma de insinuaciones o abierto regaño sobre “estar poniendo en riesgo” a su bebé, sin ser esto cierto. 

De hecho, es notable cómo en los partos domiciliarios^57^, por ejemplo, la percepción de autodeterminación, libertad y no culpabilización de las madres es siempre enfatizada, ligada además a la libertad de movimientos, el comportamiento corporal (gritar si se quiere, ir al baño si se quiere, comer o beber, practicar sexo en compañía o de forma autónoma, etc.); todo eso que en el análisis de Shabot y Korem^56^ se vincula con el cuerpo de la parturienta como “sucio”, “demasiado sexual” y “no lo suficientemente femenino”, frente al cuerpo reeducado de la “buena madre-mujer-pasiva-femenina”, que hace lo que le dicen, tema que Loughlin *et al*.^58^ abordan desde la filosofía en relación con la clínica con relación al estigma, el respeto y la culpa.

Como señalan Shabot y Korem^56^, es notable que suele plantearse a estas madres “desabridas” el bienestar de su bebé como una prioridad, debiendo ser ellas madres altruistas, frente a sus preferencias que, en teoría, ponen en riesgo el bienestar de su bebé, como si ambos bienestares fueran dos cosas diferentes (no siéndolo en realidad), presentando la realidad como un juego suma-cero, desde esa perspectiva occidental individualista, tan falaz, de la madre y su criatura como dos elementos separados, cuando sus intereses seguramente y casi siempre son confluyentes de forma intrínseca y estructural, como arriba ya se apuntó.

Esto se vincula directamente con un análisis anterior de Shabot^59^ sobre la violencia obstétrica en general como violencia estructural y de género, donde profundiza en su análisis fenomenológico primordial acerca de los sentimientos de la opresión encarnada, así como de percepción de infantilización por parte de las parturientas, justificando cómo y por qué este fenómeno es diferente de otros tipos de violencia médica, de objetivación y de cosificación. 

Siguiendo el análisis de Young sobre la existencia femenina bajo el patriarcado, Shabot^59^ explica cómo las mujeres parturientas (sus cuerpos en trabajo de parto) son potencialmente percibidos como la antítesis del mito de la feminidad; ya que, frente a su debida pasividad, resultan poderosamente activos y (re) productivos, de modo que socavan el mandato del comportamiento corporal pasivo, amenazando seriamente, por tanto, las potencias hegemónicas del patriarcado. La pasividad que se logra a través de la violencia obstétrica domesticaría los cuerpos para volverlos “femeninos” en el sentido opresivo tradicional (en contra del parto en casa, donde la mujer puede hacer lo que quiera y se llega a optar por este, literalmente, para eludir la violencia obstétrica^57^^,^^60^. Este potencial insurgente del parto en particular y la maternidad en general se revela de forma poderosísima en los movimientos de madres lactivistas, entre otros^32^.

Ello se vincula también, en gran medida, con el análisis de Ballesteros^61^, que pone el acento en cómo impacta positivamente en cualquier proceso de salud el poder involucrarse e influir en la toma de decisiones, como parte elemental del ejercicio de la autonomía, lo que suponen factores cruciales también para una experiencia de parto satisfactoria. Así, esta autora describe lo que se ha llamado el “dilema estigmatizante”, relacionado con la percepción del parto como muy riesgoso, por un lado, y la injusticia testimonial sobre las mujeres de parto, por otro, frente al corolario de la autoridad médica como máxima fuente de conocimiento y legitimidad en el proceso. Por tanto, si no *obedecen*, las mujeres podrían caer bajo el estigma de la irracionalidad o el del egoísmo (en la línea dicotómica señalada arriba por Shabot).

Chadwick^62^^,^^63^ se ocupa, en línea similar a Shabot, de la violencia obstétrica como una práctica de silenciamiento y marginalización, y cómo el mismo concepto resulta favorecedor de un léxico contestatario, siéndolo en sí mismo. Explica cómo el término de “violencia obstétrica” ha surgido como una importante herramienta, entre el reclamo legal y el activismo, en la búsqueda global de una atención obstétrica y materna, en general, humana, equitativa y respetuosa. 

Esta autora considera la violencia obstétrica no solo como un modo de descripción o noción jurídica, sino que constituye una “intervención epistémica”, en tanto que cuestiona la normalización de palabras y prácticas considerados no deseables; afirma, pues, que

…el lenguaje de la violencia obstétrica también constituye un rechazo de los marcos epistémicos que silencian, disminuyen, borran y devalúan formas alternativas y encarnadas de conocimiento reproductivo y agencia. [Traducción libre de: *the language of obstetric violence also constitutes a refusal of epistemic frames that silence, diminish, erase, and devalue alternative and embodied forms of reproductive knowledge and agency*.]^63^

Shabot^64^^,^^65^, finalmente, centra también su análisis en la forma de injusticia epistémica denominada *testimonial*, que se ejerce de forma sistemática sobre las mujeres parturientas, y en cómo a estas literalmente “no se las cree” en la sala de partos (ni antes ni después) debido a un doble prejuicio: uno, derivado simplemente de su condición de mujer; otro, de su condición de sujeto frente a la autoridad médica, lo que supone también en gran medida una doble discriminación susceptible de ser analizada desde una perspectiva interseccional^66^.

En relación con ello, siguiendo el ejemplo autorreferencial de Shabot y ya para terminar, debo decir que una de las cosas que más me sorprendió (víctima yo entonces, aún, del paradigma de patriarcado gineco-obstétrico), hablando con mis matronas para parir en casa, fue cuando les preguntaba: *“¿Y cómo sabré cuándo tengo que empujar? ¿Me avisáis?*”, y ellas siempre afirmaban: “*Tú lo vas a saber*”. Y así fue. Ellas preguntaban si lo notaba, y me decían que empujara cuando *yo sintiera que así debía ser.* Creo que ha habido pocas experiencias de empoderamiento más grandes en mi vida que aquella, la de “*empuja cuando quieras*”, para *nacer* a mis criaturas.

## REFLEXIONES CONCLUSIVAS

### La insurrección conceptual, o los conceptos desabridos

El mundo era tan reciente, que muchas cosas carecían de nombre, y para mencionarlas había que señalarlas con el dedo.^67^

Las únicas verdades que sirven son instrumentos que luego hay que tirar.^68^

Una pregunta fundamental que surge siempre, a colación de la violencia obstétrica y la supuesta no intencionalidad de sus perpetradores, es realmente por qué hay tantas buenas personas realizando malas prácticas^30^^,^^31^. Es muy importante por ello insistir en la condición sistémica y paradigmática del patriarcado, que hace pasar por normales y aceptables ciertas prácticas, incluso inadvertidas. No se trata de acusar a personas concretas, o no siempre, al menos, ni judicializar cualquier proceso.

Existe un concepto muy iluminador en filosofía que acude en nuestra ayuda para entender esto. Lo acuñó Hannah Arendt^69^, filósofa judía que, como tantos, tuvo que exilarse para sobrevivir, muchos años apátrida por convicción, en su caso. Arendt desarrolla su idea a raíz de encontrar que tantas buenas personas, educadas, nobles vecinos y padres de familia, habían colaborado con el régimen nazi e incluso operado como piezas capitales del genocidio, como fue el caso de Adolf Eichmann (un funcionario de alto nivel en el Tercer Reich).

El concepto es la *banalidad del mal*^69^. En un sistema enfermo, perverso de manera estructural en alguna o muchas de sus prácticas, el mal sucede de manera natural, sin darnos cuenta, incluso porque tenemos que cooperar con él para salvar la propia vida y mantener la homeostasis social, a la que tiende cualquier persona o colectivo. Esto ha sido observado en muchos contextos, por ejemplo, en la Sudáfrica del apartheid. El sabio Nelson Mandela^70^ hizo la misma constatación cuando trabó amistad con sus carceleros afrikáners en la Isla de Robben, racistas para con él y a la vez buenas personas en lo cotidiano.

Tenemos que cambiar muchos elementos de ese sistema patriarcal (aquel magma biopolítico), *desmovilizar*, para que el mal no suceda incluso a menudo vehiculado a través de buenas personas y buenos profesionales.

Hemos querido enfatizar especialmente el trabajo sobre el concepto de violencia obstétrica acerca de su óptica crítica en tanto injusticia epistémica, desde la fenomenología feminista y la posible pertinencia, al fin, o no, de su uso como tal. Pensemos que los conceptos son escoplos cognitivos, como afirma la filosofía: *herramientas para pensar*, pero también para *impeler a la acción*. Para el caso de la violencia obstétrica, podemos considerarlo lo que se llama en filosofía un concepto moral denso (frente a lo que sería un concepto ético estrecho), con elementos tanto descriptivos como evaluativos simultáneamente^71^. Cuando usamos el término “violencia obstétrica” no solo estamos *describiendo* algo, sino que estamos asumiendo que es algo evitable, algo rechazable. La subversión es consustancial a la palabra. Por ello quiero hablar de la violencia obstétrica como un concepto insurrecto, una noción desabrida. 

Considero que es fundamental que se sostenga el término específico de violencia obstétrica, bien que sea discutiéndolo, matizándolo, complejizándolo como decíamos, todo lo que haga falta, en ese juego de legitimidades y voces múltiples que lo ocupan y balean; no para generar nuevas y espurias dicotomías, juegos de suma-cero entre los dos supuestos bandos en disputa (la clase médica o profesional *versus* el pueblo llano, en este caso las mujeres y sus criaturas), ya que ello es falaz, sino para comprender mejor las cosas. Hay un gran poder en la enunciación, en la narración, en cómo (nos) contamos los cuentos. El potencial insurgente del parto es capital, igual que lo es en la lactancia, porque el parto no es ni mucho menos solo un acto médico o un suceso biológico, como no lo es (*no solo*) la especie humana. *Parimos biografías*, he dicho en alguna otra ocasión^72^.

El presidente del Colegio de Médicos temía que, con la propagación o consolidación de este concepto (que de todos modos ya es irrecusable), se pueda minar la confianza de la ciudadanía en la clase sanitaria, una situación de desconfianza médico-paciente, dijo él… Pero la realidad es que esa figura que todavía se suele denominar “paciente”, cada vez más, desde los paradigmas sociosanitarios más críticos y la propia bioética^23^^,^^24^, no es *solo* paciente sino también *agente*, agente de su propia salud, actora social, desde y con una comprensión de la salud no solo física, como hace ya muchas décadas viene defendiendo la propia OMS. Y tanto el médico como el paciente -por usar sus mismos términos- no son criaturas angélicas, en ningún sentido, sino actores sociales atravesados por la biopolítica, el biopoder, en todos sus aspectos, y por tanto susceptibles de agencia, error y decisión, entre otras muchas virtualidades. 

Me resulta curiosa (y también inquietante) la profunda simetría, la analogía que encontramos en el exceso de intervención (medicalización, abuso tecnológico) en la violencia obstétrica, y la deriva del mundo en términos genéricos, en la afectación sistémica de los ecosistemas que hoy llamamos cambio climático y que ha llevado a una auténtica emergencia global, al riesgo de nuestra propia vida. Ese abuso del planeta -precisamente por causa del *exceso* de civilización, tal y como ha sido entendido hasta ahora desde el mito ilustrado del progreso y su comprensión capitalista neoliberal (frente a, por ejemplo, el decrecentismo)- asume su corolario en cómo sucede el parto entre los seres humanos. En esta coyuntura, una reflexión, más que ética, *bioética* en un sentido genuino^23^^,^^24^, se hace perentoria e irrenunciable. Y es que no en vano la violencia obstétrica ha sido ya reclamada como asunto de salud pública global (dada su notoria prevalencia) y de alarmante gravedad^73^.

Tenemos que quitar las bridas a los conceptos, si hace falta. Por eso hablamos de palabras desabridas. Tenemos que dejarlas hacer su propia insurrección. Para poder continuar transitando del “negacionismo sanitario, académico y mediático al diálogo”^11^. Por un paradigma de salud y de atención a la salud mucho más vasto, integrador, decolonial y, desde luego, antipatriarcal, es fundamental usar el término “violencia obstétrica”, sin ambages ni temor, mientras nos hallemos en aquel interregno señalado al inicio; seguir usándolo, precisamente, para significarlo y atribuirlo políticamente, legitimarlo epistémicamente y, sobre todo, conjurarlo práctica y activamente. 

A las barricadas.
